# A Challenging Case of Pseudohyperkalemia in Chronic Lymphocytic Leukemia

**DOI:** 10.1177/2324709617746194

**Published:** 2017-12-13

**Authors:** Muhamad Alhaj Moustafa, Vera Malkovska, Sherif Elmahdy, Joseph Catlett

**Affiliations:** 1 MedStar Washington Hospital Center, Washington, DC, USA

**Keywords:** pseudohyperkalemia, chronic lymphocytic leukemia, whole blood potassium, leukocytosis, hyperkalemia

## Abstract

Pseudohyperkalemia is an uncommon finding in chronic lymphocytic leukemia. It is a misleading condition that could lead to iatrogenic hypokalemia when unwarranted treatment is administered. We describe an interesting case of pseudohyperkalemia in severe leukocytosis and how we identified it.

## Introduction

Pseudohyperkalemia is a marked increase in serum potassium level in vitro in the absence of any clinical or electrocardiogram evidence for hyperkalemia. It is most frequently seen as a result of red cell hemolysis, which is easily recognized in the laboratory. It is also seen in patients with thrombocytosis and/or leukocytosis. It was first described in 1955 by Hartmann and Mellinkoff, who noted cases of pseudohyperkalemia but no associated symptoms in samples from patients with thrombocytosis.^1^ In 1975, Bellevue et al reported 2 cases of pseudohyperkalemia in severe leukocytosis.^2^

Pseudohyperkalemia is not well recognized in patients with chronic lymphocytic leukemia (CLL) due to its infrequent incidence. This represents a challenging medical dilemma due to the possibility of inducing severe hypokalemia after unwarranted treatment. In this article, we discuss a case of pseudohyperkalemia in a CLL patient with severe leukocytosis.

## Case Description

A 52-year-old male with a known diagnosis of CLL presented to the emergency medicine department with worsening fatigue and dyspnea on exertion over the preceding 2 weeks. Laboratory studies showed an elevated white blood cell (WBC) count at 537 × 10^9^/L, Hgb 2.8 g/dL, and platelet count 22 × 10^9^/L. The patient had CLL RAI stage IV with severe anemia, thrombocytopenia, extensive lymphadenopathy, and hepatosplenomegaly. The patient had not been previously treated for CLL. The first potassium level check occurred in the emergency department laboratory where the blood sample was delivered by hand within 30 minutes of collection and with no pneumatic tube transportation (potassium level was 5 mmol/L). The following potassium checks were on the medicine wards in which a pneumatic tube transportation was used. Potassium was elevated on multiple reads (Table 1). The patient had no clinical evidence of hyperkalemia. Frequent electrocardiogram monitoring showed no sign of hyperkalemia. It was thought that the level was falsely elevated due to break down of WBCs due to technical problem when drawing blood. On day 3, arterial sample and venous sample (both done by the physician to ensure correct techniques and prompt delivery to the laboratory) were collected at the same time using a serum vacutainer. Both were analyzed at the same time and within 30 minutes from blood draw. Elevated potassium was reported in both samples with no hemolysis. A whole blood potassium sample was collected (done by same physician) with an arterial blood gas (ABG) heparinized syringe showing a normal potassium level. Repeatedly, over the patient’s hospital stay, whole blood potassium samples collected (collected by different physicians) by using an ABG heparinized syringe showed a normal potassium level regardless of time to sample analysis (Table 1). The patient received a temporizing therapy for hyperkalemia, which was discontinued immediately after confirming pseudohyperkalemia. The patient was treated with BR (bendamustine and rituximab) instead of FCR (fludarabine, cyclophosphamide, and rituximab) due concern of exacerbating anemia and thrombocytopenia as a side effect of fludarabine. After cycle one, WBCs decreased to 12 × 10^9^/L. By the time of submitting this article, the patient is status post 3 cycles of BR with normalization of WBC, Hgb, and platelet counts. Potassium levels normalized on outpatient checks.

**Table 1.** Potassium Levels by Specimen and Transport Method^a^.

Day	Time (Hours)	Specimen Type	Transport Method	Potassium Level^b^ (mmol/L)
1	01:38	Venous, Serum vacutainer	Walked	5
1	21:56	Venous, Serum vacutainer	Tubed	6.9
2	00:36	Venous, Serum vacutainer	Tubed^c^	4.9
2	16:35	Venous, Serum vacutainer	Tubed	>10.0
2	20:17	Venous, Serum vacutainer	Tubed	6.8
2	22:17	Venous, Serum vacutainer	Tubed	6.3
3	00:40	Venous, Serum vacutainer	Tubed	7.4
3	04:09	Venous, Serum vacutainer	Tubed	5.6
3	13:46	Venous, Serum vacutainer	Tubed^c^	>10.0
3	13:46	Arterial, Serum vacutainer	Tubed^c^	8.2
3	16:09	Whole blood, ABG	Tubed^c^	3.9
4	08:26	Venous, Serum vacutainer	Tubed	5.1
4	22:18	Venous, Serum vacutainer	Tubed	6.5
5	05:23	Venous, Serum vacutainer	Tubed	5.7
5	11:17	Venous, Serum vacutainer	Tubed	5.9
6	04:01	Venous, Serum vacutainer	Tubed	7.0
6	06:32	Whole blood, ABG	Tubed	4.0
6	12:30	Venous, Serum vacutainer	Tubed	8.3
6	18:14	Venous, Serum vacutainer	Tubed	8.9
7	05:06	Venous, Serum vacutainer	Tubed	6.4
8	01:05	Whole blood, ABG	Tubed	4.6
8	06:01	Venous, Serum vacutainer	Tubed	5.4

Abbreviation: ABG, arterial blood gas.

a None of the samples was hemolyzed.

b Normal range = 3.5-5.1.

c Time to analysis <30 minutes.

## Discussion

Although true severe hyperkalemia is a life-threatening condition that requires immediate intervention, pseudohyperkalemia is a false elevation in in-vitro potassium level and requires no intervention. However, a misleading falsely elevated level of potassium can lead to inappropriate treatment and potential fatal outcomes due to medically induced hypokalemia. In general, pseudohyperkalemia can happen during the collection process (difficult venipuncture, small-size needles, using vacutainers causing negative pressure and destruction of the cells, excessive shaking of the sample, and using hand fist maneuver, etc), transport (pneumatic transportation, slow delivery to laboratory, etc), storage of specimens (prolonged storage, cold storage, etc), or while processing the specimen (centrifugation of the sample may cause potassium leak due to cell destruction, especially of abnormal cell walls, or during blood coagulation process, etc).^3^ A careful evaluation of hyperkalemia in patients with severe leukocytosis is crucial. Early recognition of the condition can prevent unwarranted treatments and unwanted complications.

In our patient a temporizing treatment was initiated (pending further testing) with Kayexalate and dextrose 50% with 10 international units of insulin. There was a concern that he might develop tumor lysis syndrome due to his tumor burden and elevated potassium level might be the first sign. The patient was not placed on dialysis due to high suspicion of pseudohyperkalemia. We hypothesized that pseudohyperkalemia in this case was due to multiple factors: (*a*) negative pressure in vacutainer caused destruction of the fragile leukemic blast cells; (*b*) the large number of WBCs might exaggerate the leakage effect of potassium due to coagulation from cells in nonheparinized specimens; and (*c*) serum samples underwent centrifugation, which might have led to extensive blasts destruction as opposed to whole blood potassium samples. Other possible contributing factors for pseudohyperkalemia include increased usage of limited metabolic resources by large number of WBCs leading to impaired sodium/potassium adenosine triphosphatase activity and the release of potassium from cells, and mechanical trauma secondary to pneumatic tube transportation.^4^

This case highlights the importance of considering pseudohyperkalemia in cases with severe leukocytosis to avoid iatrogenic hypokalemia and its detrimental consequences. The use heparinized ABG kits for whole blood potassium could prevent the coagulation of the blood and the extensive release of potassium from the leukemic cells. Pneumatic tube transport systems are a potential cause of pseudohyperkalemia, especially in patients with high WBCs. However, samples collected using heparinized blood gas syringe were not affected in this case. Decreasing the time between blood collection and potassium level testing might be helpful, although not evident in our case.
